# Paradoxical Effects of Fruit on Obesity

**DOI:** 10.3390/nu8100633

**Published:** 2016-10-14

**Authors:** Satya P. Sharma, Hea J. Chung, Hyeon J. Kim, Seong T. Hong

**Affiliations:** 1 Department of Biomedical Sciences and Institute for Medical Science, Chonbuk National University Medical School, Jeonju 54907, Korea; satyapriya83@gmail.com; 2 Department of Microbiology, Seonam University Medical School, Namwon 55724, Korea; hjchung@chonbuk.ac.kr; 3 JINIS BDRD Institute, JINIS Biopharmaceuticals Co., 948-9 Dunsan, Bongdong, Wanju 55321, Korea; hjkim@jinisbio.com

**Keywords:** obesity, fruit, anti-obesity, pro-obesity

## Abstract

Obesity is exponentially increasing regardless of its preventable characteristics. The current measures for preventing obesity have failed to address the severity and prevalence of obesity, so alternative approaches based on nutritional and diet changes are attracting attention for the treatment of obesity. Fruit contains large amounts of simple sugars (glucose, fructose, sucrose, etc.), which are well known to induce obesity. Thus, considering the amount of simple sugars found in fruit, it is reasonable to expect that their consumption should contribute to obesity rather than weight reduction. However, epidemiological research has consistently shown that most types of fruit have anti-obesity effects. Thus, due to their anti-obesity effects as well as their vitamin and mineral contents, health organizations are suggesting the consumption of fruit for weight reduction purposes. These contradictory characteristics of fruit with respect to human body weight management motivated us to study previous research to understand the contribution of different types of fruit to weight management. In this review article, we analyze and discuss the relationships between fruit and their anti-obesity effects based on numerous possible underlying mechanisms, and we conclude that each type of fruit has different effects on body weight.

## 1. Introduction

The significant enhancement in food production during the agriculture revolution in the 18th and early 19th centuries has resolved the problem of famine to an extent, which had constantly threatened the survival of the human species, but paradoxically, modern humans are living in an era when easy access to the energy-dense food is a concern because continuous intake of an energy-dense diet positively influences the body–energy equilibrium and can cause obesity. Throughout the world, modern societies are faced with the problems of obesity and obesity-related diseases. According to the World Health Organization (WHO), worldwide obesity has doubled since 1980. At present, >1.9 billion adults and >42 million children under the age of 5 years are overweight worldwide, indicating its prevalence in all age groups [1].

Obesity is a multifactorial disease caused by biological, behavioral, and environmental factors [2,3,4,5], but it is mainly attributed to low physical activity and high consumption of energy-dense food for a prolonged period [6]. Obesity is now one of the main problems in modern society, and intensive research is currently underway to search for a solution to obesity [7]. Current management of obesity has been done at the individual level as well as at the community level. The individual preventions for obesity are mostly based on pharmacosurgical interventions with little success. The outcomes of pharmaceutical drug use for weight management are not significant, and the long-term consumption of these anti-obesity agents might have severe side effects [8]. Surgical manipulations are only useful in the most extreme cases [9]. In addition to the individual-base, population- or community-level intervention for obesity has been pursued by modifying behavioral factors like increasing physical activities, reduction in sedentary lifestyle, healthy diet, and so forth [10,11,12]. Policies and environmental changes also contribute to weight management. In this context, increasing fruit and vegetable intake is widely recommended for preventing and/or treating obesity [13,14,15].

The beneficial health effects of fruit are well established [16,17]. The consumption of fruit is known to attenuate obesity and obesity-related diseases such as diabetes and coronary heart disease [18,19,20,21,22]. Similar to the western population, fruit consumption has a dose-response relationship with cardiovascular disease in Asia [23,24]. However, in contrast to fruit intake, there is no significant association between the intake of vegetables and hypertriglyceridemia [23,24]. Furthermore, several meta-analyses have provided strong evidence that higher consumption of fruit and vegetables is associated with a lower risk of all-causes mortality, including cardiovascular disease and cancer [25,26,27,28]. Thus, low fruit consumption is considered to be the fourth leading contributor to the global disease burden, and thus one of the major attributable risk factors for diseases such as being overweight (high body-mass index (BMI)), hyperglycemia, and hypercholesterolemia [29]. 

Clearly, fruit has beneficial effects on health through its anti-obesity effects. Many clinical studies have shown that increasing the daily consumption of fruit is inversely correlated to weight gain [30]. It was also shown that the consumption of whole fruit contributes to a reduced risk of long-term weight gain in adults by reducing the total energy intake [31]. Several mechanisms are thought to be responsible for the anti-obesity effect produced by fruit, but still it is tough to point out a particular mechanism that allows some simple sugar-rich fruits to contribute to anti-obesity. Most types of fruit have very high simple sugar content, such as sucrose, fructose, glucose, etc. [32]. Thus, considering that the overconsumption of simple sugars is one of the main causes of obesity and related diseases [33,34,35], it is perhaps surprising that the consumption of fruit is associated with anti-obesity in most cases. For this review, we searched available health sciences electronic database, including MEDLINE, Science Direct, Google Scholar, and the Web of Science in two independent phases. First, the paper selection was made exclusively for epidemiological studies to support the anti-obesity and pro-obesity effects of fruit published in the last 20 years, from 1996 to June 2016. Combination of following keywords was used in the process of paper selection: fruit, whole fruit, fruit juice, canned fruit, dried fruit, obesity, body weight, weight gain, BMI, and waist circumference. Second, the search was performed for gathering the information to support anti-obesity and pro-obesity mechanisms, which had no time limitation. Here, we included studies that showed the link between fruit consumption and obesity, thereby providing insights into how simple sugar-rich fruits may contribute to anti-obesity.

## 2. Anti-Obesity Effect of Fruits

**Figure 1:**
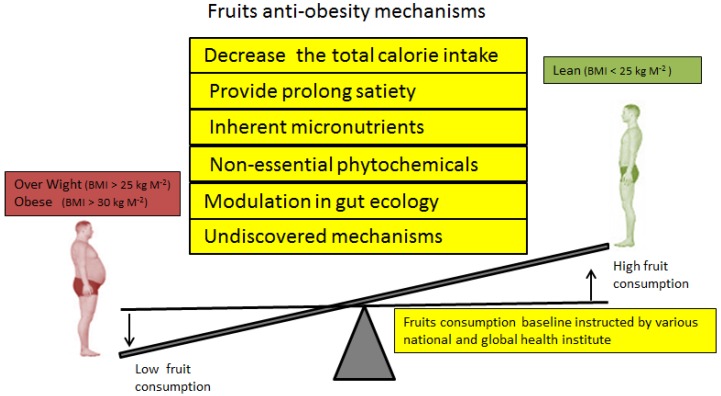
Higher consumption of daily fruit is recommended by health organizations as a key factor for maintaining a healthy body weight via various mechanisms.

Numerous interventional and observational human trials based on longitudinal and cross-section study designs ranging from small to large population sets in various countries have investigated the close association between the consumption of fruit and obesity. Based on precise anthropometric analyses related to obesity, such as body weight, BMI, and waist circumference (WC), the majority of these studies have suggested that fruit intake is inversely associated with obesity, as shown in Figure 1. These human studies of the association between fruit and obesity can be broadly classified into three different categories: (i) intervention randomized clinical trials (IRCTs); (ii) prospective cohort studies; and (iii) cross-section studies.

### 2.1. Intervention Randomized Clinical Trials (IRCTs)

IRCTs conducted in obese and overweight individuals have shown that fruit intake significantly suppresses obesity [36,37,38,39,40]. The first of these studies reported the impact of low and high fruit diets on the body weight, BMI, and WC of obese Spanish females with a BMI of 34.9 ± 2.3 kg·m^−2^ and average age of 32.6 ± 5.8 years [36]. After 8 weeks of fruit intervention, there were significant reductions in all parameters in both groups, but a comparative analysis only detected a significant difference in WC. Fujioka et al. reported another IRCT in American obese individuals with a mean BMI of 35.6 ± 4.7 kg·m^−^^2^ and a broad age range of 18–65 years, a larger sample size (*n* = 77), and longer follow-up [37]. This was a four-armed double-blinded placebo trial with three intervention groups, who consumed extra fresh grapefruit, grapefruit juice, and apple juice. The body weight was reduced in all groups, but the parameters in the group who consumed fresh grapefruit were significantly different compared with the placebo. Another study with apple and pear intake in a hypocaloric diet found significant reductions in body weight and BMI compared with eating oat cookies after 10 weeks [38]. In this IRCT, three groups of obese Brazilian women with a BMI of 31.9 ± 4.2 kg·m^−2^ and average age of 44.1 ± 5.4 years received 300 g/day of either apple or pear as a fruit in their diet, whereas the control group consumed 60 g/day oat cookies for 10 weeks. In another IRTC where the original aim was to evaluate the effect of mangosteen juice on obesity biomarkers in American middle-aged obese population, increased mangosteen juice intake for 8 weeks significantly reduced the BMI and body fat mass [39]. A recent intervention study found that pomegranate juice intake significantly reduced fat accumulation, but the changes in body weight and BMI were not clinically significant [40]. Finally, the outcomes of intervention studies conducted in obese individuals have shown that fruit intake ameliorates obesity-associated parameters, e.g., reduced body weight, improved BMI, and decreased WC. Considering that the intervention studies mentioned above were conducted for short time periods, the impact of fruit intake on obesity appears to be very significant.

### 2.2. Prospective Observational Studies

Prospective human studies also support the inverse association between fruit and obesity, where these studies have reported data obtained from obese or overweight populations as well as comparisons with normal weight individuals. Most of the studies do not specify the factors that were controlled in the trails. However, the individuals with severe illnesses such as metabolic disorders, terminal diseases, cardiovescular diseases, and other serious illnesses that can influnece the end results were not included in these studies; however, smoking, drinking, physical activity, diet intake, etc. were not restricted but were monitered in the course of the studies. In 1997, Stamler et al. reported that a higher amount of energy intake in the form of fruit resulted in greater weight loss [41]. In this study, 5%–6% of the total energy provided as whole fruit or fruit juice was considered to be responsible for an annual average weight loss of 2.3–6.8 kg. He et al. conducted a 12-year prospective study in a large population of borderline overweight middle-aged female nurses (*n* = 74,063) with a BMI of 24.9 kg·m^−2^ and an average age of 50.7 ± 7 years [42]. They reported that an increased amount of fruit in the diet reduced the likelihood of obesity. They stated that 0.22 increments and 1.86 increments in fruit servings per day reduced the risk of obesity by up to 14% and 24%, respectively. Vioque et al. also reported an inverse correlation between higher whole fruit intake and weight gain [43]. Buijsse et al. reported that an increase of 100 g per day in the whole fruit intake was correlated with an average weight loss of 0.017 kg in the overweight population based on data collected from five European countries [44]. Rautiainen et al. tested the relationship between fruit intake and weight gain in postmenopausal normal-weight women, thereby demonstrating that a greater intake of fruit alone reduced the risk of becoming obese [45]. A meta-analysis of 17 independent prospective studies reported by Schwingshackl also suggested that the intake of fruit has an inverse association with weight gain [46]. Other prospective studies have also found negative associations between fruit intake and weight gain or obesity [47,48,49,50,51,52]. Most of these prospective studies indicated that fruit intake may be linked with body weight maintenance among normal weight or marginally overweight individuals. These studies also showed that the increases in body weight were slightly lower in a relatively high fruit intake study group compared with those who consumed less fruit. 

### 2.3. Cross-Sectional Studies

Several cross-sectional studies support the inverse relationship between the amounts of fruit consumed and weight gain over a period of time [53,54,55,56,57]. The first cross-sectional study published in the USA by Serdula et al. in 1996 considered a large population of adult men and women (*n* = 21,892; men = 9292, women = 12,599; age > 18 years). This study showed similar fruit consumption rates in all of the different body weight groups, where the results indicated no association between fruit intake and body weight [53]. After two years in 1998, Trudeau et al. also found no association between fruit consumption and BMI [54]. In 2002, Lin and Morrison performed a survey of a large adult population in the USA (*n* = 9117, male = 4709, female = 4408, age > 19) and found a negative association between fruit consumption and body weight [55]. After four years in 2008, Moreira and Padrao also reported a negative association between fruit and obesity, but only in women and not in men [56]. In the same year, Davis et al. reported a negative relationship between daily fruit intake and body weight [57]. Two of these studies found inverse correlations only in female individuals and not in the male population [54,56]. However, the results reported from most of these studies of large populations including males and females detected a negative relationship between fruit consumption and obesity, but self-reporting of end results by the participants were used to analyze the final outcome in most of cases, thereby reducing the reliability of the final results.

## 3. Probable Mechanisms for the Anti-Obesity Effect of Fruit

**Figure 2:**
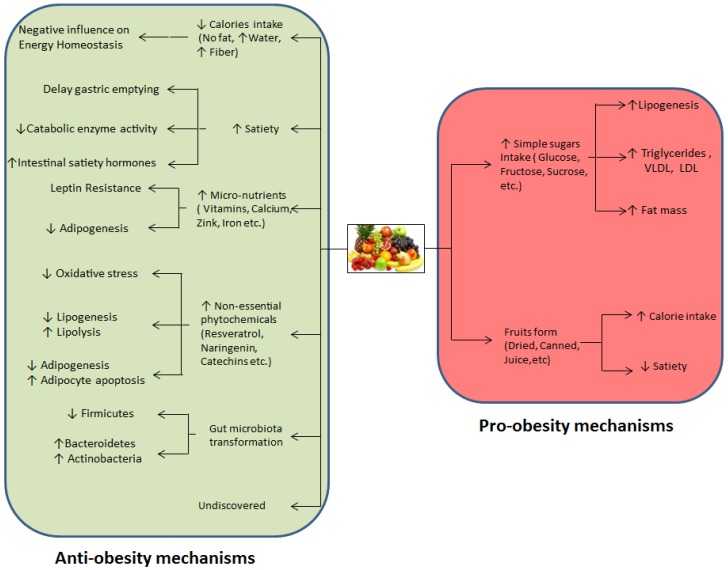
Possible mechanisms responsible for the anti-obesity or pro-obesity effects of fruits.

As it is known that multiple mechanisms are responsible for producing the complex obesity condition, thus, only one specific mechanism cannot be considered exclusively for obesity reduction or restricting weight gain by fruit consumption. In particular, multiple concepts or mechanisms may be responsible for the anti-obesity effects of fruit. Figure 2 shows the possible mechanisms related to the anti-obesity effects of fruit, which are discussed in the following paragraphs.

### 3.1. Fruit Reduces Calorific Consumption

In daily diet, replacing the energy-dense food with increased amount of fruit appears to be inversely correlated with weight gain. The underlying mechanism responsible for the anti-obesity effect of fruit is not clearly understood. One logical explanation for weight reduction by fruit consumption may be a decrease in the total energy intake and a consequent amelioration of energy disequilibrium. Energy homeostasis plays a key role in obesity generation, where a chronic positive disequilibrium in energy homeostasis is thought to be an underlying cause that generates pathophysiological changes, thereby resulting in obesity. A positive influence on energy homeostasis increases the growth of adipose tissue for storing excess energy, which accelerates the growth of the persistent fat mass, thereby resulting in the excessive accumulation of adipose tissue in the condition of obesity [58]. Fruit is considered a low-energy-dense food based on two features. First, most types of fruit contain negligible amounts of fat, which is the major energy-producing macronutrient and the main contributor to obesity generation [59]. Second, fruit contains a large amount of water and a considerable amount of dietary fiber, which may be responsible for further diluting the energy density [16,20]. Thus, fruit provides a lower amount of energy per unit compared with westernized processed food, which can negatively affect the energy balance [60]. Therefore, the addition of fruit to the daily diet reduces overall energy consumption and improves the energy disequilibrium. Thus, the continuous intake of fruit may restrict weight gains, reduce the fat mass, and control obesity.

### 3.2. Fruit Provides Prolonged Satiety

Satiety is a physiological process, which regulates the appetite or hunger by secreting several biochemical signal peptides from various parts of the body [61]. Food enriched with dietary fiber leads to an extended satiety state, which can reduce the gross food intake and directly influence the total energy consumption [62]. Dietary fiber is known to create a viscous gel-like environment in the small intestine, which delays gastric emptying and reduces the activity of enzymes responsible for digesting carbohydrate, fat, and protein. Moreover, this slower digestion of energy-generating macronutrients increases the nutrient-receptor communication for a prolonged period, so the secretion of intestinal satiety hormones may subsequently reduce the hunger sensation and finally decrease the consumption of food [63,64]. In addition, the gel produced by soluble fiber increases the volume of undigested food and reduces the amount of calories extracted, thereby reducing the total energy acquired from the diet [65,66,67]. Nearly all types of fruit contain high amounts of dietary fiber, and if they are consumed whole, they can enhance the satiety state and reduce feelings of hunger [68].

### 3.3. Fruit Micronutrients Influence Obesity-Associated Metabolic Pathways

Micronutrients are essential nutritional elements, which are required in very small amounts to maintain good health but their deficiency can cause various health conditions, including metabolic disorders. Micronutrient deficiencies are correlated with obesity through various mechanisms [69]. For instance, low concentrations of vitamins have been reported in obese populations in several studies [70,71,72,73]. In particular, vitamins A, E, and C are negatively associated with fat deposition or central obesity [74,75,76]. This influence of vitamins on fat mass reduction is attributed to leptin resistance and the downregulation of genes involved in adipocyte generation and differentiation [77]. In addition to vitamins, minerals are crucial micronutrients when considering obesity in general. Minerals such as zinc [78,79], iron [80,81], and calcium [82] have inverse correlations with fat generation or obesity in animal studies as well as in human trials. Thus, to reduce the incidence of obesity, the micronutrient concentration should be maintained at the desired level in the body by natural or formulated resources. Fruit is one of the most endowed and accessible providers of essential micronutrients among natural foods [16,20]. Thus, the consumption of fruit can provide essential micronutrients to limit obesity via various mechanisms. Therefore, the presence of various micronutrients in different types of fruit could be one of the underlying mechanisms responsible for their anti-obesity effect. However, further studies are required to provide direct evidence for the anti-obesity effects of micronutrients in fruits.

### 3.4. Non-Essential Phytochemicals in Fruit Augment Their Anti-Obesity Effects

Numerous phytochemicals are found in fruit, where they are typically byproducts of multiple plant metabolic pathways or synthesized exclusively for multiple protective mechanisms, such as combating infectious diseases, surviving under environmental stress conditions, and due to excessive exposure to ultraviolet radiation [17]. Most fruit phytochemicals are not essential for our vital functions or human nutrients. However, they have desirable health benefits by decreasing the risk of multiple illnesses, such as cancers, type 2 diabetes, cardiovascular diseases, and obesity [83,84]. In particular, fruit phytochemicals explicitly have anti-obesity effects, according to numerous studies, by beneficially altering multiple physiological cascades, e.g., reducing oxidative stress via their anti-oxidant properties, suppressing adipogenesis, inhibiting the differentiation of preadipocytes, stimulating lipolysis, initiating the apoptosis of adipocytes, and limiting lipogenesis [85]. Plant phytochemicals can be categorized into six main classes, but fruit phytochemicals are primarily phenolic compounds. Specific isolated phenolic compounds such as resveratrol, caffeic acid, naringenin, proanthocyanidins, catechins, and cyanidin are known anti-obesity compounds found in fruit [17,85,86,87,88]. A previous study performed phenolic phytochemical profiling in 25 of the most commonly consumed fruits, and found that each fruit contained unique phenolic constituents in substantial amounts as well as in proportion to others [89]. Among the 25 types of fruit, wild blue- and blackberries accumulated the highest phenolic phytochemical contents, followed by pomegranate, cranberry, blueberry, plum, and apple. Obviously, the beneficial health effects of the abundant phytochemicals found in fruit may contribute synergistically to the anti-obesity effects of fruit consumption.

### 3.5. Effects of Fruit on Gut Microbial Ecology

Human gut microbes are thought to affect health [90] by modulating the metabolic phenotype [91]. In particular, the microbial community of obese individuals is surprisingly distinct from that of their non-obese or lean counterparts [92]. This change in the gut microbial ecosystem may be affected by several internal and external factors [93,94,95,96,97]. Diet is the main daily external source and the key factor that can influence the gut microbial ecology rapidly and substantially [98,99]. The dietary components that influence the gut microbial ecology are enriched in dietary fiber, and polyphenols can increase the ratios of the bacterial phyla Bacteroidetes and Actinobacteria, which are predominant in lean individuals, but decrease the prevalence of Firmicutes and Proteobacteria, which are dominant in the obese gut community [100,101,102]. Therefore, the incorporation of fruit in the diet drives the gut ecology toward an anti-obese condition by increasing the prevalence of lean-type bacteria but reducing that of obese-type bacteria. At present, insufficient studies have investigated the direct relationship between fruit consumption and changes in the gut microbial community. However, it was recently reported that the phylum Bacteriodetes was abundant in a fruit-consumption group who exhibited significant reductions in body weight [103]. Similarly, increasing the fruit content of children’s meals led to increases in the bacterial phyla Bacteroidetes and Actinobacteria, which are related to lean individuals [104]. More studies are required of the detailed mechanisms responsible for the modulatory effects of the gut microbial ecology on host obesity and the incorporation of fruit in the diet to modulate the beneficial effects of the gut microbial ecology.

### 3.6. Undiscovered Mechanisms

Fruit are complex biochemical products of plants. The detailed compositions of various fruits are known, but the biochemical compositions of each fruit are not well understood [16,105]. Every year, new types of phytochemicals are reported in different fruits with various beneficial health properties [17]. In addition, the complexity of obesity is well known because various impaired molecular and physiological pathways are involved in the establishment of the pathophysiological state of obesity. Therefore, given the unknown biochemicals in fruit and their relationship with obesity, various mechanisms are probably undiscovered, and it will take several years before they are elucidated.

## 4. Probable Mechanisms for the Pro-Obesity Effect of Fruit

### 4.1. Fruits with Pro-Obesity Effects

Despite the recognized anti-obesity effect of fruit, several studies have also demonstrated the pro-obesity effects of certain types of fruit. An early study was conducted in preschool-aged children [106] as a cross-sectional trial in the USA, which analyzed the impact of various dietary components on the health and growth of infants and young children. The comparative statistical analysis performed in this study showed that higher fruit juice consumption in children significantly increased the probability of becoming obese as well as lower height. According to the dietary guidelines in the USA, 8 ounces of 100% fruit juice contain most phytochemicals and almost as much as the whole fruit, but it excludes dietary fiber [107]. Thus, the outcomes of this study contradicted the current understanding of the beneficial health effects of fruit and fruit products. Six years later, Field et al. provided supported for this earlier investigation based on a prospective cohort study of a group of pre-adolescent and adolescent boys and girls [108]. Their analysis based on 15,000 American children suggested that the consumption of a fruit-rich diet could explain weight gains in the preadolescent and adolescent age groups if the total calorific intake was not adjusted according to their requirements. In 2006, Rush et al. conducted a randomized controlled study and found that a high kiwi fruit intake increased the body weight compared with non-consumers of the same fruit [109]. In the same year, another study found a positive association between fruit juice consumption and adiposity in children aged 1 to 4 years [110]. However, increased adipose tissue mass was found only in the already obese or overweight children, and increased fruit juice intake only augmented the risk of weight gain or obesity. In 2007, Libuda et al. reported on the ongoing longitudinal DONALD study of German adolescent boys and girls. Their statistical analysis showed that fruit juice consumption was positively associated with BMI in girls, but there was only a cross-sectional association in boys [111]. In 2011, Makkes et al. also reported a positive association between fruit juice intake and BMI in school-going children aged 8–10 years based on cross-sectional trials conducted in Guatemala. The consumption of fruit juice, either homemade or commercially produced, resulted in a significantly higher BMI in children [112]. In 2015, Shefferly et al. reported the results of a longitudinal study conducted in a large group of U.S. children aged 2–5 years, which suggested that greater consumption of fruit juices on a regular basis was correlated with a higher increase in BMI with age [113]. 

However, the final outcomes of many of these human trials regarding fruit intake and its correlation with weight support the pro-obesity effects of certain fruits. Many epidemiological studies have confirmed the pro-obesity effects of various fruits, but the precise mechanisms responsible for obesity development due to fruit consumption are not yet established. Therefore, we discuss some factors that may be responsible for the weight gain effects of consuming these fruits, as shown in Figure 2.

### 4.2. Fruit Increases Simple Sugars Intake

Among the various possible pro-obesity factors related to fruit, the high concentration of fruit sugars is considered to be the main factor responsible for weight gain via the increased consumption of some fruit. A higher daily intake of sugars is associated with an increased risk of metabolic disorders, specifically obesity [114,115,116], according to many epidemiological and animal studies [117,118,119]. In particular, simple sugars, such as glucose and fructose, are thought to be the main source for generating fatty acids by hepatic de novo lipogenesis, which may increase the levels of hepatic and circulatory triglycerides, as well as the circulating levels of very-low- and low-density lipoproteins, thereby resulting in increases in adipose tissue and obesity [120,121,122]. Most fruits are well known to be rich sources of naturally occurring sugars, particularly simple sugars such as glucose, sucrose, fructose, etc. [32]. Therefore, consuming an increased proportion of fruit containing higher amounts of sugars in the daily diet may directly increase the ingestion of simple sugars to positively influence the body energy equilibrium. This positive impact on the body energy equilibrium due to the increased consumption of fruit rich in simple sugars may lead to obesity over a long period. Thus, the consumption of certain types of fruit that are rich in simple sugars is the most likely mechanism for the association between obesity and fruit consumption. 

### 4.3. Improper Form of Fruit Supplements with Pro-Obesity Effects

Commercialization of food increases the availability of ready-to-eat fruit that excels the accessibility of exotic fruits as well as increase the shelf-life. Commercially processed fruits are available in various forms like dried, canned, juices, etc. However, fruit juice often contains similar or more nutrients compared to whole fruit, but significant reduction in dietary fibers reduce the satiety state and enhance the hunger feeling, contributing to additional intake of foods [123]. Canned fruit, most of the time, contains a high amount of simple sugar for taste and/or conservation purposes. This supplement of simple sugar to fruits increases calorie intake. Dried fruits contain a negligible amount of water, which makes them energy-dense compared to the whole fruit. Processed fruits contain more sugars, less fiber or less water, making them high-energy-dense food compared to whole fruits [124]. Hence, replacing whole fruit with any common processed fruit type could be a possible contributor to the obesity.

## 5. Discussion

Over the past 30 years, many studies have investigated the effects of fruit consumption on obesity. Most of these studies support the inverse correlation between fruit intake and unhealthy weight gain or obesity, irrespective of the study type. As mentioned earlier, various health organizations have stated that the amount of fruit in the daily diet has a negative relationship with weight gain (Figure 1), but there is still insufficient concrete evidence to establish a convincing mechanism, so the benefits of fruit consumption are in a confused state. However, various underlying explanations have been suggested for obesity reduction by fruit consumption, as shown in Figure 2, but the low-energy density of fruit is widely accepted as having a negative impact on the body energy balance. Fruit can negatively influence energy homeostasis via two basic mechanisms: by providing less energy per serving and reducing the daily food intake by extending satiety. Positive energy homeostasis is the fundamental cause of obesity, so both mechanisms can help to reduce the total energy inflow and ameliorate this disequilibrium in energy homeostasis by influencing it negatively. This lower energy intake increases the requirement for calories to meet the body’s daily requirements. Therefore, to compensate for the required daily amount of calories, the body utilizes stored energy from white adipose tissue, and this negative flow of energy from adipose tissue lowers the fat mass. Hence, a steady intake of fruit can reduce central obesity as well as preventing weight gain, thereby controlling obesity. 

In addition to correcting defective energy homeostasis, the second main anti-obesity mechanism related to fruit involves the essential and nonessential phytochemicals provided by fruit. Fruit are natural providers of essential required micronutrients such as vitamins and minerals, which are also responsible for producing anti-obesity effects via various mechanisms, e.g., reducing the proliferation of adipocytes and differentiation, which ultimately decreases adipogenesis and controls obesity. Therefore, the abundance of essential micronutrients in fruit may also be responsible for their anti-obesity effects. In addition to micronutrients, fruits accumulate abundant amounts of various phytochemicals, which are not essential bodily requirements, but they also contribute to the control of obesity. These phytochemicals influence the expression levels of various genes, which can repair the impaired pathophysiological cascades involved with obesity generation, e.g., by reducing oxidative stress, controlling hyperlipidemia by enhancing lipolysis and decreasing lipogenesis, and decreasing the fat mass by reducing adipogenesis and increasing adipoapoptosis. Thus, fruit phytochemicals may also augment the anti-obesity properties of fruit, although they are not essential bodily requirements. The concentrations of anti-obesity-related elements are very low in fruit so more human trials are required to draw any conclusions regarding the roles of essential and nonessential fruit phytochemicals in the control of obesity.

Finally, a very new concept related to fruit and their anti-obesity effects is that fruit can modify the gut microbial community. However, the association between gut microbes and obesity is a recent concept, although it is widely accepted because of the distinct gut microbial communities found in lean and obese individuals. The predominant bacterial phylum found in lean people may reduce energy absorption and maintain a healthy body weight. Several animal trials have shown that a steady intake of fruit can transform the gut ecology so it matches that found in a lean population. Therefore, this may be a crucial mechanism involved with the weight reduction process due to increased daily fruit intake. Thus, we have summarized various mechanisms related to fruit and their anti-obesity effects, but it is not possible to suggest that a single mechanism is responsible for weight control or obesity reduction. Indeed, all of mechanisms described above may work synergistically to produce the anti-obesity effects of fruit consumption, as shown in Figure 2.

By contrast, several studies support the pro-obesity effects of fruit, and a few mechanisms are thought to be responsible for generating obesity via fruit intake, as described in Figure 2. In particular, the abundance of simple sugars in fruit is the main direct factor that is considered to be responsible. Thus, fruit accumulates simple sugars rather than fat, but this can still be important, particularly fructose. High fructose concentrations in food are directly associated with many metabolic disorders, especially obesity via de novo lipogenesis. In addition, consuming fruit in different forms other than whole fruit can increase the calorific intake and positively influence energy homeostasis to eventually promote obesity. Thus, the generation of obesity or weight gain by consuming large amount of fruit or fruit products is an avoidable problem. Likewise, fruit and fruit products contribute to the obesity; it might be possible that fruit can also influence the metabolically healthy obesity (MHO) [125].

The results of several human studies support the pro-obesity effects of fruit, but the reported trials have various limitations such as a lack of total daily energy intake records for participants or the self-reporting of fruit intake by participants, which make their results debatable. In addition, it should be noted that the pro-obesity effects are often limited to specific age groups in children or preadolescent individuals. Moreover, studies that considered fruit juices and processed fruits in an energy-dense form should be distinguished from those of unprocessed fruit. A large number of population-based studies support the anti-obesity effects of fruit but several animal studies provide experimental proof of an inverse relationship between body weight and specific types of fruit or fruit groups via various mechanisms, such as altering the expression of various genes involved in obesity generation or reducing the appetite. No previous animal trials have tested the pro-obesity effects of fruit. Indeed, despite the high sugar content of any type of fruit, some fruits produce anti-obesity effects, thereby indicating that the anti-obesity mechanisms of fruits can suppress the pro-obesity effects by working synergistically in order to reduce obesity. Furthermore, some types of fruit have weight-reducing or weight-restricting effects on obese people, but the mechanisms responsible for these effects are still not well understood. Therefore, various fruits may have other weight-reducing components that remain undiscovered. Thus, further experiments are required to discover the mechanisms related to the unidentified anti-obesity factors in these types of fruit. Hence, the anti-obesity effects of fruit are more obvious than their pro-obesity effects. The possible mechanisms of weight reduction due to fruit consumption, e.g., low-energy-dense food, satiety factor production, and the effects of micronutrients on metabolic pathways, have indirect correlations with obesity and the intake of fruit, but they still have no direct support, particularly in humans. Therefore, extensive experiments are required to establish robust mechanisms for the anti-obesity effects of fruit.

## 6. Conclusions

Previous studies of the effects of fruit on obesity have shown that their anti-obesity effects are greater than their pro-obesity effects in most cases, as demonstrated in this review. Moreover, the final outcomes of these anti-obesity studies support the inclusion of higher amounts of fruit in our daily food intake to reduce weight, as well as promote a healthy life style by increasing physical activity and reducing the intake of sugar and fat. However, most types of fruit contain large amounts of simple sugars, which are well-known contributory factors to obesity and obesity-related diseases. Therefore, the high level intake of particular forms of fruit such as fruit juice is not advisable in certain age groups, especially children. In addition, it has been suggested that plant fiber and phytochemicals may be responsible for the anti-obesity effects of fruit, but cereals are also rich in phytochemicals and fiber yet they have no remarkable anti-obesity effects. Therefore, more human trials are required to address these two mechanisms. In addition to the possible anti-obesity mechanisms discussed above, we consider that various other important unknown components present in fruit may be responsible for preventing obesity. Furthermore, the anti-obesity properties of known fruit components need to be verified. Thus, future research should be focusing on identifying anti-obesity components in fruit as an urgent and important task in order to understand the scientific mechanism of obesity but also to develop a method for controlling obesity by increasing fruit consumption.

## References

[1] World Health Organisation Fact Sheet N°311. http://www.who.int/mediacentre/factsheets/fs311/en/.

[2] Keith S.W., Redden D.T., Katzmarzyk P.T., Boggiano M.M., Hanlon E.C., Benca R.M., Ruden D., Pietrobelli A., Barger J.L., Fontaine K.R. (2006). Putative contributors to the secular increase in obesity: Exploring the roads less traveled. Int. J. Obes. (Lond.).

[3] Janesick A.S., Shioda T., Blumberg B. (2014). Transgenerational inheritance of prenatal obesogen exposure. Mol. Cell. Endocrinol..

[4] Swinburn B.A., Sacks G., Hall K.D., McPherson K., Finegood D.T., Moodie M.L., Gortmaker S.L. (2011). The global obesity pandemic: Shaped by global drivers and local environments. Lancet.

[5] Gregor M.F., Hotamisligil G.S. (2011). Inflammatory mechanisms in obesity. Annu. Rev. Immunol..

[6] Hall K.D., Sacks G., Chandramohan D., Chow C.C., Wang Y.C., Gortmaker S.L., Swinburn B.A. (2011). Quantification of the effect of energy imbalance on bodyweight. Lancet.

[7] Bray G.A., Frühbeck G., Ryan D.H., Wilding J.P. (2016). Management of obesity. Lancet.

[8] Ryan D., Heaner M. (2014). Guidelines (2013) for managing overweight and obesity in adults. Preface to the full report. Obesity (Silver Spring).

[9] Mechanick J.I., Youdim A., Jones D.B., Garvey W.T., Hurley D.L., McMahon M.M., Heinberg L.J., Kushner R., Adams T.D., Shikora S. (2013). Clinical practice guidelines for the perioperative nutritional, metabolic, and nonsurgical support of the bariatric surgery patient—2013 update: Cosponsored by American Association of Clinical Endocrinologists, The Obesity Society, and American Society for Metabolic & Bariatric Surgery. Obesity (Silver Spring).

[10] Kumanyika S.K., Obarzanek E., Stettler N., American Heart Association Council on Epidemiology, Prevention, Interdisciplinary Committee for Prevention (2008). Population-based prevention of obesity: The need for comprehensive promotion of healthful eating, physical activity, and energy balance: A scientific statement from American Heart Association Council on Epidemiology and Prevention, Interdisciplinary Committee for Prevention (formerly the expert panel on population and prevention science). Circulation.

[11] Sacks F.M., Bray G.A., Carey V.J., Smith S.R., Ryan D.H., Anton S.D., McManus K., Champagne C.M., Bishop L.M., Laranjo N. (2009). Comparison of weight-loss diets with different compositions of fat, protein, and carbohydrates. N. Engl. J. Med..

[12] Hu T., Mills K.T., Yao L., Demanelis K., Eloustaz M., Yancy W.S., Kelly T.N., He J., Bazzano L.A. (2012). Effects of low-carbohydrate diets versus low-fat diets on metabolic risk factors: A meta-analysis of randomized controlled clinical trials. Am. J. Epidemiol..

[13] Huo R., Du T., Xu Y., Xu W., Chen X., Sun K., Yu X. (2015). Effects of Mediterranean-style diet on glycemic control, weight loss and cardiovascular risk factors among type 2 diabetes individuals: A meta-analysis. Eur. J. Clin. Nutr..

[14] Boeing H., Bechthold A., Bub A., Ellinger S., Haller D., Kroke A., Leschik-Bonnet E., Müller M.J., Oberritter H., Schulze M. (2012). Critical review vegetables and fruit in the prevention of chronic diseases. Eur. J. Nutr..

[15] Centers for Disease Control and Prevention (2011). Strategies to Prevent Obesity and Other Chronic Diseases: The CDC Guide to Strategies to Increase the Consumption of Fruits and Vegetables.

[16] Slavin J.L., Lloyd B. (2012). Health benefits of fruits and vegetables. Adv. Nutr..

[17] Liu R.H. (2013). Health-promoting components of fruits and vegetables in the diet. Adv. Nutr..

[18] Tohill B.C., Seymour J., Serdula M., Kettel-Khan L., Rolls B.J. (2004). What epidemiologic studies tell us about the relationship between fruit and vegetable consumption and body weight. Nutr. Rev..

[19] Rolls B.J., Ello-Martin J.A., Tohill B.C. (2004). What can intervention studies tell us about the relationship between fruit and vegetable consumption and weight management?. Nutr. Rev..

[20] Devalaraja S., Jain S., Yadav H. (2011). Exotic Fruits as Therapeutic Complements for Diabetes, Obesity and Metabolic Syndrome. Food Res. Int..

[21] Montonen J., Knekt P., Jarvinen R., Reunanen A. (2004). Dietary antioxidant intake and risk of type 2diabetes. Diabetes Care.

[22] Fung T.T., Chiuve S.E., McCullough M.L., Rexrode K.M., Logroscino G., Hu F.B. (2008). Adherence to a DASH-style diet and risk of coronary heart disease and stroke in women. Arch. Intern. Med..

[23] Du H., Li L., Bennett D., Guo Y., Key T.J., Bian Z., Sherliker P., Gao H., Chen Y., Yang L. (2016). China kadoorie biobank study. Fresh fruit consumption and major cardiovascular disease in China. N. Engl. J. Med..

[24] Yuan C., Lee H.J., Shin H.J., Stampfer M.J., Cho E. (2015). Fruit and vegetable consumption and hypertriglyceridemia: Korean National Health and Nutrition Examination Surveys (KNHANES) 2007–2009. Eur. J. Clin. Nutr..

[25] He F.J., Nowson C.A., MacGregor G.A. (2006). Fruit and vegetable consumption and stroke: Meta-analysis of cohort studies. Lancet.

[26] World Cancer Research Fund/American Institute for Cancer Research (2007). Food, Nutrition, Physical Activity, and the Prevention of Cancer: A Global Perspective.

[27] Wang X., Ouyang Y., Liu J., Zhu M., Zhao G., Bao W., Hu F.B. (2014). Fruit and vegetable consumption and mortality from all causes, cardiovascular disease, and cancer: Systematic review and dose-response meta-analysis of prospective cohort studies. BMJ.

[28] Oyebode O., Gordon D.V., Walker A., Mindell J.S. (2014). Fruit and vegetable consumption and all-cause, cancer and CVD mortality: Analysis of Health Survey for England data. J. Epidemiol. Community Health.

[29] Lim S.S., Vos T. (2012). A comparative risk assessment of burden of disease and injury attributable to 67 risk factors and risk factor clusters in 21 regions, 1990–2010: A systematic analysis for the Global Burden of Disease Study 2010. Lancet.

[30] Alinia S., Hels O., Tetens I. (2009). The potential association between fruit intake and body weight—A review. Obes. Rev..

[31] Hebden L., O’Leary F., Rangan A., Singgih L.E., Hirani V., Allman F.M. (2015). Fruit Consumption and Adiposity Status in Adults: A Systematic Review of Current Evidence. Crit. Rev. Food Sci. Nutr..

[32] Lee J. (2015). Sorbitol, Rubus fruit, and misconception. Food Chem..

[33] Van-Dam R.M., Seidell J.C. (2007). Carbohydrate intake and obesity. Eur. J. Clin. Nutr..

[34] Bosy W.A., Müller M.J. (2015). Impact of carbohydrates on weight regain. Curr. Opin. Clin. Nutr. Metab. Care.

[35] Buyken A.E., Goletzke J., Joslowski G., Felbick A., Cheng G., Herder C., Brand-Miller J.C. (2014). Association between carbohydrate quality and inflammatory markers: Systematic review of observational and interventional studies. Am. J. Clin. Nutr..

[36] Rodríguez M.C., Parra M.D., Marques L.I., de Morentin B.E., González A., Martínez J.A. (2005). Effects of two energy-restricted diets containing different fruit amounts on body weight loss and macronutrient oxidation. Plant Foods Hum. Nutr..

[37] Fujioka K., Greenway F., Sheard J., Ying Y. (2006). The effects of grapefruit on weight and insulin resistance: Relationship to the metabolic syndrome. J. Med. Food.

[38] De Oliveira M.C., Sichieri R., Venturim Mozzer R. (2008). A low-energy-dense diet adding fruit reduces weight and energy intake in women. Appetite.

[39] Udani J.K., Singh B.B., Barrett M.L., Singh V.J. (2009). Evaluation of Mangosteen juice blend on biomarkers of inflammation in obese subjects: A pilot, dose finding study. Nutr. J..

[40] González O.M., Martínez A.E., Espinel-Bermúdez M.C., Pérez-Rubio K.G. (2011). Effect of pomegranate juice on insulin secretion and sensitivity in patients with obesity. Ann. Nutr. Metab..

[41] Stamler J., Dolecek T.A. (1997). Relation of food and nutrient intakes to body mass in the special intervention and usual care groups in the Multiple Risk Factor Intervention Trial. Am. J. Clin. Nutr..

[42] He K., Hu F.B., Colditz G.A., Manson J.E., Willett W.C., Liu S. (2004). Changes in intake of fruits and vegetables in relation to risk of obesity and weight gain among middle-aged women. Int. J. Obes. Relat. Metab. Disord..

[43] Vioque J., Weinbrenner T., Castelló A., Asensio L., Garcia de la Hera M. (2008). Intake of fruits and vegetables in relation to 10-year weight gain among Spanish adults. Obesity (Silver Spring).

[44] Buijsse B., Feskens E.J., Schulze M.B., Forouhi N.G., Wareham N.J., Sharp S., Palli D., Tognon G., Halkjaer J., Tjønneland A. (2009). Fruit and vegetable intakes and subsequent changes in body weight in European populations: Results from the project on Diet, Obesity, and Genes (DiOGenes). Am. J. Clin. Nutr..

[45] Rautiainen S., Wang L., Lee I.M., Manson J.E., Buring J.E., Sesso H.D. (2015). Higher Intake of Fruit, but Not Vegetables or Fiber, at Baseline Is Associated with Lower Risk of Becoming Overweight or Obese in Middle-Aged and Older Women of Normal BMI at Baseline. J. Nutr..

[46] Schwingshackl L., Hoffmann G., Kalle-Uhlmann T., Arregui M., Buijsse B., Boeing H. (2015). Fruit and Vegetable Consumption and Changes in Anthropometric Variables in Adult Populations: A Systematic Review and Meta-Analysis of Prospective Cohort Studies. PLoS ONE.

[47] Schulz M., Kroke A., Liese A.D., Hoffmann K., Bergmann M.M., Boeing H. (2002). Food groups as predictors for short-term weight changes in men and women of the EPIC-Potsdam cohort. J. Nutr..

[48] Drapeau V., Després J.P., Bouchard C., Allard L., Fournier G., Leblanc C., Tremblay A. (2004). Modifications in food-group consumption are related to long-term body-weight changes. Am. J. Clin. Nutr..

[49] Nooyens A.C., Visscher T.L., Schuit A.J., van Rossum C.T., Verschuren W.M., Van Mechelen W., Seidell J.C. (2005). Effects of retirement on lifestyle in relation to changes in weight and waist circumference in Dutch men: A prospective study. Public Health Nutr..

[50] Linde J.A., Utter J., Jeffery R.W., Sherwood N.E., Pronk N.P., Boyle R.G. (2006). Specific food intake, fat and fiber intake, and behavioral correlates of BMI among overweight and obese members of a managed care organization. Int. J. Behav. Nutr. Phys. Act..

[51] Sánchez-Villegas A., Bes-Rastrollo M., Martínez-González M.A., Serra-Majem L. (2006). Adherence to a Mediterranean dietary pattern and weight gain in a follow-up study: The SUN cohort. Int. J. Obes. (Lond.).

[52] Te Velde S.J., Twisk J.W., Brug J. (2007). Tracking of fruit and vegetable consumption from adolescence into adulthood and its longitudinal association with overweight. Br. J. Nutr..

[53] Serdula M.K., Byers T., Mokdad A.H., Simoes E., Mendlein J.M., Coates R.J. (1996). The association between fruit and vegetable intake and chronic disease risk factors. Epidemiology.

[54] Trudeau E., Kristal A.R., Li S., Patterson R.E. (1998). Demographic and psychosocial predictors of fruit and vegetable intakes differ: Implications for dietary interventions. J. Am. Diet. Assoc..

[55] Lin B.H., Morrison R.M. (2002). Higher fruit consumption linked with lower body mass index. Food Rev..

[56] Moreira P., Padrao P. (2006). Educational, economic and dietary determinants of obesity in Portuguese adults: A cross-sectional study. Eat. Behav..

[57] Davis J.N., Hodges V.A., Gillham M.B. (2006). Normal-weight adults consume more fiber and fruit than their age- and height-matched overweight/obese counterparts. J. Am. Diet. Assoc..

[58] Hill J.O., Wyatt H.R., Peters J.C. (2012). Energy balance and obesity. Circulation.

[59] U.S. Food and Drug Administration. http://www.fda.gov/downloads/Food/IngredientsPackagingLabeling/LabelingNutrition/UCM169225.pdf.

[60] Mozaffarian D., Hao T., Rimm E.B., Willett W.C., Hu F.B. (2011). Changes in diet and lifestyle and long-term weight gain in women and men. N. Engl. J. Med..

[61] Blundell J., de Graaf C., Hulshof T., Jebb S., Livingstone B., Lluch A., Mela D., Salah S., Schuring E., van der Knaap H. (2010). Appetite control: Methodological aspects of the evaluation of foods. Obes. Rev..

[62] Halford J.C., Harrold J.A. (2012). Satiety-enhancing products for appetite control: Science and regulation of functional foods for weight management. Proc. Nutr. Soc..

[63] French S.J., Read N.W. (1994). Effect of guar gum on hunger and satiety after meals of differing fat content: Relationship with gastric emptying. Am. J. Clin. Nutr..

[64] Baer D.J., Rumpler W.V., Miles C.W., Fahey G.C. (1997). Dietary fiber decreases the metabolizable energy content and nutrient digestibility of mixed diets fed to humans. J. Nutr..

[65] Burton F.B. (2000). Dietary fiber and energy regulation. J. Nutr..

[66] Spiller G.A. (2001). CRC Hand Book of Dietary Fiber in Human Nutrition.

[67] Lairon D., McCleary B.V., Prosky L. (2001). Dietary fiber, dietary lipids. Advanced Dietary Fiber Technology.

[68] Flood-Obbagy J.E., Rolls B.J. (2009). The effect of fruit in different forms on energy intake and satiety at a meal. Appetite.

[69] García O.P., Long K.Z., Rosado J.L. (2009). Impact of micronutrient deficiencies on obesity. Nutr. Rev..

[70] Aasheim E.T., Hofsø D., Hjelmesaeth J., Birkeland K.I., Bøhmer T. (2008). Vitamin status in morbidly obese patients: A cross-sectional study. Am. J. Clin. Nutr..

[71] Galan P., Viteri F.E., Bertrais S., Czernichow S., Faure H., Arnaud J., Ruffieux D., Chenal S., Arnault N., Favier A. (2005). Serum concentrations of beta-carotene, vitamins C and E, zinc and selenium are influenced by sex, age, diet, smoking status, alcohol consumption and corpulence in a general French adult population. Eur. J. Clin. Nutr..

[72] Myara I., Alamowitch C., Michel O., Heudes D., Bariety J., Guy-Grand B., Chevalier J. (2003). Lipoprotein oxidation and plasma vitamin E in nondiabetic normotensive obese patients. Obes. Res..

[73] Reitman A., Friedrich I., Ben A.A., Levy Y. (2002). Low plasma antioxidants and normal plasma B vitamins and homocysteine in patients with severe obesity. Isr. Med. Assoc. J..

[74] Vaughan L.A., Benyshek D.C., Martin J.F. (1997). Food acquisition habits, nutrient intakes, and anthropometric data of Havasupai adults. J. Am. Diet. Assoc..

[75] Johnston C.S., Beezhold B.L., Mostow B., Swan P.D. (2007). Plasma vitamin C is inversely related to body mass index and waist circumference but not to plasma adiponectin in nonsmoking adults. J. Nutr..

[76] Viroonudomphol D., Pongpaew P., Tungtrongchitr R., Changbumrung S., Tungtrongchitr A., Phonrat B., Vudhivai N., Schelp F.P. (2003). The relationships between anthropometric measurements, serum vitamin A and E concentrations and lipid profiles in overweight and obese subjects. Asia Pac. J. Clin. Nutr..

[77] Aeberli I., Molinari L., Spinas G., Lehmann R., l’Allemand D., Zimmermann M.B. (2006). Dietary intakes of fat and antioxidant vitamins are predictors of subclinical inflammation in overweight Swiss children. Am. J. Clin. Nutr..

[78] Arsenault J.E., Havel P.J., López de Romaña D., Penny M.E., Van Loan M.D., Brown K.H. (2007). Longitudinal measures of circulating leptin and ghrelin concentrations are associated with the growth of young Peruvian children but are not affected by zinc supplementation. Am. J. Clin. Nutr..

[79] Padmavathi I.J., Kishore Y.D., Venu L., Ganeshan M., Harishankar N., Giridharan N.V., Raghunath M. (2009). Prenatal and perinatal zinc restriction: Effects on body composition, glucose tolerance and insulin response in rat offspring. Exp. Physiol..

[80] Pinhas-Hamiel O., Newfield R.S., Koren I., Agmon A., Lilos P., Phillip M. (2003). Greater prevalence of iron deficiency in overweight and obese children and adolescents. Int. J. Obes. Relat. Metab. Disord..

[81] Lecube A., Carrera A., Losada E., Hernández C., Simó R., Mesa J. (2006). Iron deficiency in obese postmenopausal women. Obesity (Silver Spring).

[82] Villarroel P., Villalobos E., Reyes M., Cifuentes M. (2014). Calcium, obesity, and the role of the calcium-sensing receptor. Nutr. Rev..

[83] Scalbert A., Manach C., Morand C., Rémésy C., Jiménez L. (2005). Dietary polyphenols and the prevention of diseases. Crit. Rev. Food Sci. Nutr..

[84] Pandey K.B., Rizvi S.I. (2009). Plant polyphenols as dietary antioxidants in human health and disease. Oxid. Med. Cell. Longev..

[85] Meydani M., Hasan S.T. (2010). Dietary polyphenols and obesity. Nutrients.

[86] Wang S., Moustaid-Moussa N., Chen L., Mo H., Shastri A., Su R., Bapat P., Kwun I., Shen C.L. (2014). Novel insights of dietary polyphenols and obesity. J. Nutr. Biochem..

[87] Rupasinghe H.P., Sekhon-Loodu S., Mantso T., Panayiotidis M.I. (2016). Phytochemicals in regulating fatty acid β-oxidation: Potential underlying mechanisms and their involvement in obesity and weight loss. Pharmacol. Ther..

[88] Amiot M.J., Riva C., Vinet A. (2016). Effects of dietary polyphenols on metabolic syndrome features in humans: A systematic review. Obes. Rev..

[89] Wolfe K.L., Kang X., He X., Dong M., Zhang Q., Liu R.H. (2008). Cellular antioxidant activity of common fruits. J. Agric. Food Chem..

[90] Guarner F., Malagelada J.R. (2003). Gut flora in health and disease. Lancet.

[91] Li M. (2008). Symbiotic gut microbesmodulate human metabolic phenotypes. Proc. Natl. Acad. Sci. USA.

[92] Turnbaugh P.J., Hamady M., Yatsunenko T., Cantarel B.L., Duncan A., Ley R.E., Sogin M.L., Jones W.J., Roe B.A., Affourtit J.P. (2009). A core gut microbiome in obese and lean twins. Nature.

[93] Albenberg L.G., Wu G.D. (2014). Diet and the intestinal microbiome: Associations, functions, and implications for health and disease. Gastroenterology.

[94] Walker A.W., Ince J., Duncan S.H. (2011). Dominant and diet-responsive groups of bacteria within the human colonic microbiota. ISME J..

[95] Hooper L.V., Littman D.R., Macpherson A.J. (2012). Interactions between the microbiota and the immune system. Science.

[96] Cotter P.D., Stanton C., Ross R.P., Hill C. (2012). The impact of antibiotics on the gut microbiota as revealed by high throughput DNA sequencing. Discov. Med..

[97] Clarke S.F., Murphy E.F., O’Sullivan O. (2014). Exercise and associated dietary extremes impact on gut microbial diversity. Gut.

[98] Wu G.D., Chen J., Hoffmann C. (2011). Linking long-term dietary patterns with gut microbial enterotypes. Science.

[99] Scott K.P., Gratz S.W., Sheridan P.O., Flint H.J., Duncan S.H. (2013). The influence of diet on the gut microbiota. Pharmacol. Res..

[100] Simpson H.L., Campbell B.J. (2015). Review article: Dietary fibre-microbiota interactions. Aliment. Pharmacol. Ther..

[101] Parkar S.G., Stevenson D.E., Skinner M.A. (2008). The potential influence of fruit polyphenols on colonic microflora and human gut health. Int. J. Food Microbiol..

[102] Tzounis X., Vulevic J., Kuhnle G.G., George T., Leonczak J., Gibson G.R., Kwik-Uribe C., Spencer J.P. (2008). Flavanol monomer-induced changes to the human faecal microflora. Br. J. Nutr..

[103] Noratto G.D., Garcia-Mazcorro J.F., Markel M., Martino H.S., Minamoto Y., Steiner J.M., Byrne D., Suchodolski J.S., Mertens-Talcott S.U. (2014). Carbohydrate-free peach (*Prunus persica*) and plum (*Prunus salicina)* [corrected] juice affects fecal microbial ecology in an obese animal model. PLoS ONE.

[104] De Filippo C., Cavalieri D., di Paola M., Ramazzotti M., Poullet J.B., Massart S., Collini S., Pieraccini G., Lionetti P. (2010). Impact of diet in shaping gut microbiota revealed by a comparative study in children from Europe and rural Africa. Proc. Natl. Acad. Sci. USA.

[105] Liu R.H. (2003). Health benefits of fruit and vegetables are from additive and synergistic combinations of phytochemicals. Am. J. Clin. Nutr..

[106] Dennisocn B.A., Rockwell H.L., Baker S.L. (1997). Excess fruit juice consumption by preschool-aged children is associated with short stature and obesity. Pediatrics.

[107] U.S. Department of Agriculture, U.S. Department of Health and Human Services (2010). Dietary Guidelines for Americans.

[108] Field A.E., Gillman M.W., Rosner B., Rockett H.R., Colditz G.A. (2003). Association between fruit and vegetable intake and change in body mass index among a large sample of children and adolescents in the United States. Int. J. Obes. Relat. Metab. Disord..

[109] Rush E., Ferguson L.R., Cumin M., Thakur V., Karunasinghe N., Plank L. (2006). Kiwifruit consumption reduces DNA fragility: A randomized controlled pilot study in volunteers. Nutr. Res..

[110] Faith M.S., Dennison B.A., Edmunds L.S., Stratton H.H. (2006). Fruit juice intake predicts increased adiposity gain in children from low-income families: Weight status-by-environment interaction. Pediatrics.

[111] Libuda L., Alexy U., Sichert-Hellert W., Stehle P., Karaolis-Danckert N., Buyken A.E., Kersting M. (2008). Pattern of beverage consumption and long-term association with body-weight status in German adolescents—Results from the DONALD study. Br. J. Nutr..

[112] Makkes S., Montenegro B.G., Groeneveld I.F., Doak C.M., Solomons N.W. (2011). Beverage consumption and anthropometric outcomes among schoolchildren in Guatemala. Matern. Child. Nutr..

[113] Shefferly A., Scharf R.J., DeBoer M.D. (2016). Longitudinal evaluation of 100% fruit juice consumption on BMI status in 2–5-year-old children. Pediatr. Obes..

[114] Rippe J.M., Angelopoulos T.J. (2016). Added sugars and risk factors for obesity, diabetes and heart disease. Int. J. Obes. (Lond.).

[115] Bray G.A., Popkin B.M. (2014). Dietary sugar and body weight: Have we reached a crisis in the epidemic of obesity and diabetes?. Diabetes Care.

[116] Lustig R.H., Schmidt L.A., Brindis C.D. (2012). Public health: The toxic truth about sugar. Nature.

[117] Bray G.A., Nielsen S.J., Popkin B.M. (2004). Consumption of high-fructose corn syrup in beverages may play a role in the epidemic of obesity. Am. J. Clin. Nutr..

[118] Te Morenga L., Mallard S., Mann J. (2012). Dietary sugars and body weight: Systematic review and meta-analyses of randomised controlled trials and cohort studies. BMJ.

[119] Kanarek R.B., Orthen-Gambill N. (1982). Differential effects of sucrose, fructose and glucose on carbohydrate-induced obesity in rats. J. Nutr..

[120] Tappy L., Lê K.A. (2010). Metabolic effects of fructose and the worldwide increase in obesity. Physiol. Rev..

[121] Hellerstein M.K., Schwarz J.M., Neese R.A. (1996). Regulation of hepatic de novo lipogenesis in humans. Annu. Rev. Nutr..

[122] Samuel V.T. (2011). Fructose induced lipogenesis: From sugar to fat to insulin resistance. Trends Endocrinol. Metab..

[123] Clemens R., Drewnowski A., Ferruzzi M.G., Toner C.D., Welland D. (2015). Squeezing fact from fiction about 100% fruit juice. Adv. Nutr..

[124] Choose MyPlate.gov (2011). Washington (DC): United States Department of Agriculture (USDA). http://www.choosemyplate.gov.

[125] Camhi S.M., Whitney Evans E., Hayman L.L., Lichtenstein A.H., Must A. (2015). Healthy eating index and metabolically healthy obesity in U.S. adolescents and adults. Prev. Med..

